# Rediscovering Recovery: Reconceptualizing Underlying Assumptions of Citizenship and Interrelated Notions of Care and Support

**DOI:** 10.1100/2012/496579

**Published:** 2012-12-23

**Authors:** Caroline Vandekinderen, Griet Roets, Rudi Roose, Geert Van Hove

**Affiliations:** ^1^Department of Special Education, Ghent University, Henri Dunantlaan 2, 9000 Ghent, Belgium; ^2^Department of Social Welfare Studies, Ghent University, Henri Dunantlaan 2, 9000 Ghent, Belgium

## Abstract

Over the last few decades, research, policy, and practice in the field of mental health care and a complementary variety of social work and social service delivery have internationally concentrated on *recovery* as a promising concept. In this paper, a conceptual distinction is made between an *individual* approach and a *social* approach to recovery, and underlying assumptions of citizenship and interrelated notions and features of care and support are identified. It is argued that the conditionality of the individual approach to recovery refers to a conceptualization of citizenship as *normative*, based on the existence of a norm that operates in every domain of our society. We argue that these assumptions place a burden of *self-governance* on citizens with mental health problems and risk producing people with mental health problems as nonrecyclable citizens. The social approach to recovery embraces a different conceptualization of citizenship as *relational and inclusive* and embodies the myriad ways in which the belonging of people with mental health problems can be constructed in practice. As such, we hope to enable social services and professionals in the field to balance their role in the provision of care and support to service users with mental health problems.

## 1. Introduction 

In the field of (mental) health care and a complementary variety of social work and social service delivery, the emergence of new understandings and paradigms of care and support for people with mental health problems has been observed over the last few decades [1–4]. Since the mid-1980s, research, policy, and practice have internationally concentrated on *recovery* as an inspiring concept [1, 5–10]. The recovery paradigm was considered to be a promising and innovative framework [6, 11] that justified the deinstitutionalization of residential services over the last few decades [12, 13] and has enabled an increasing emphasis on developing community-based services in different Western welfare states [13–19]. This development has been associated with the emergence of new ideas about citizenship, focusing on the right of people with mental health problems to live on equal terms in mainstream society and promoting social inclusion in the community [4]. As discussed by Peck et al. [20, page 442], these ideas have challenged both traditional service structures and the authority of the “professional narrative about the nature of, and responses to, mental distress.”

Quite recently it has been argued, however, that there is an urgent need for conceptual clarity about what constitutes recovery-oriented practice [9, 21], since “key knowledge gaps have been identified as the need for clarity about the underpinning philosophy of recovery” [21, page 449]. In many Western countries, the ambiguity of the emerging concept of recovery in mental health has “created major dilemmas about how to develop adequate (…) community-based services in the context of recurring financial underfunding” [19, page 426]. The central issue implies how mental health systems and services can support the recovery process [1, 2]. In this paper, based on a comprehensive review of the recovery literature and recent empirical research [22–25], a conceptual distinction is made between an *individual* approach and a *social *approach to recovery. First, we will outline the scope of the recovery paradigm. Second, underlying assumptions of citizenship and interrelated notions and features of care and support are identified in each of these approaches to recovery. As Slade [2, page 703] recently asserted, the domain of promoting citizenship among individuals in recovery “has been the least investigated, and yet, plausibly, it is the most influential. Improving social inclusion and community integration requires clinicians (and social service professions) to pay more attention to supporting the person to make connections and to the creation of inclusive communities.”

## 2. The Scope of the Recovery Paradigm

The recovery movement grew in the realms of the self-help and deinstitutionalization movement in the 1960s and 1970s, when ideas about promoting a life in the community and providing adequate care and support were increasingly developing a broad social base [5, 26, 27]. Since the mid-1980s, an impressive body of knowledge about mental health recovery has been generated from the perspectives and experiences of service users, family members, and mental health and social work professionals [21, 28–34]. The recovery paradigm rejects the assumption that being diagnosed with—even chronic—mental health problems is inevitably considered a tragic catastrophe and the cause of becoming a social outcast [35], and an attempt is made to “reach beyond our storehouse of writings that describe psychiatric disorder as a catastrophic life event” [33, page 335]. Although there are many perceptions and definitions of recovery, William Anthony, Director of the Boston Center for Psychiatric Rehabilitation, introduces a cornerstone definition of mental health recovery, identifying recovery as “a deeply personal, unique process of changing one's attitudes, values, feelings, goals, skills and/or roles. It is a way of living a satisfying, hopeful, and contributing life, even with limitations caused by illness. Recovery involves the development of new meaning and purpose in one's life as one grows beyond the catastrophic effects of mental illness” [5, page 27]. As an approach that constitutes a branch of the comprehensive family of strengths-based perspectives [36], the key themes and ingredients in the academic literature base, including published first-person recovery narrative accounts, can be identified as embracing strengths rather than weaknesses, hope rather than despair, and engagement and active participation in life rather than withdrawal and isolation [2, 6, 21, 31, 33]. At first glance, the recovery discourse explains recovery in terms of a journey of hope [31], consisting of a lifelong, individual process in which the individual takes back control, gets on with his/her life [37], and (re)integrates into the social world [38]. In a nutshell, recovery is grafted onto empowering service users with mental health problems to stimulate their personal growth and responsibility [35].

In what follows, we focus on different conceptual notions of recovery and on the complicated issues and dilemmas that are emerging concerning the ways in which care and support can be provided by professionals [13, 19], as it is stated that social service professionals play a pivotal role in supporting service users with mental health problems in their recovery [1, 2, 17]. In the extensive body of recovery literature, we identify and distinguish an *individual* and a *social* approach to recovery. In our conceptual analysis, these different conceptualizations of recovery intrinsically construct different notions of citizenship and imply disabling as well as enabling features of care and support offered by professionals in social service delivery. In the individual approach to recovery, an underlying notion of *normative *citizenship is persistently at work, implying a *residual perspective* on care and support services. In the social approach to recovery, an underlying notion of *relational and inclusive* citizenship is uncovered, enabling a *structural perspective *on care and support services.

## 3. An Individual Approach to Recovery

In both theory and practice, stressing the service user's responsibility appears to be a central component in the empowering process of recovery [39]. According to Deegan [31, page 2], for example, recovery involves enabling people with mental health problems to “regain control over their lives, and (…) be responsible for their own individual journey of recovery.” Recently, mental health experts formulated a working definition of recovery as a person-driven process: “self-determination and self-direction are the foundations for recovery as individuals define their own life goals and design their unique path(s) towards those goals. Individuals optimize their autonomy and independence to the greatest extent possible by leading, controlling, and exercising choice over the services and supports that assist their recovery and resilience. In so doing, they are empowered and provided the resources to make informed decisions, initiate recovery, build on their strengths, and gain or regain control over their lives” [40]. The majority of recovery-oriented researchers and practitioners emphasize that recovery involves a resurgence of a coherent sense of self and of personal responsibility for one's own state of being in the process of social reintegration [41, 42]. In that vein, the work of recovery-oriented professionals revolves around a logic of empowerment to stimulate personal growth [43]. Craig [44, page 126] formulates the recovery-oriented task of the services as “a matter of doing as much as possible to empower the individual. The aim is to have consumers assume more and more responsibility for themselves. Their particular responsibilities include developing goals, working with providers and others—for example, family and friends—to make plans for reaching these goals, taking on decision-making tasks, and *engaging in self-care*. In addition, responsibility is a factor in making choices and taking risks; *full empowerment requires that consumers live with the consequences of their choices*.” As Jacobson and Greenley [38, page 483] state, “empowerment emerges from inside one's self—although it may be facilitated by external conditions.”

In the most favorable and far-reaching view, the individual approach to recovery suggests that people with mental health problems individually have to take “personal responsibility through self-management, being responsible for your own well-being” [1, page 268]. As Slade [2, page 703] asserts, “the central shift in a recovery-oriented system, therefore, involves seeing an individual not as a patient—someone who is fundamentally different and therefore needs treatment before getting on with life—but as a person whose efforts to live the most fulfilling life possible are fundamentally similar to those of people without mental illness.” Nevertheless, although the recovery paradigm is heretical within the dominant biomedical model [33, 39], “the fashionable concept of ‘recovery' can be a two-edged sword” (Hopton, [12, pp. 65-66]). As Hopton [12, pp. 65-66] argues aptly, “on one level, it represents a step away from the once prevalent idea that (…) only compliance to medication will prevent a relapse. On the other hand, (sometimes) it also seems to have medical overtones.” In clinical conceptualizations, for example, it is stated that recovery implies that it is possible to regain control of one's life, to reintegrate socially and become *independent* [41], and to “return to a normal or healthy state, *free of the symptoms of illness*, (…) being able to work, to go to college, to live in ordinary housing, have an active recreational life and find friendship and romance” [44, page 125, our italics]. This clinical and diagnostic emphasis on difference and intrapsychic deficits that should be overcome by the individual who is engaging in self-care and expected to *recover* from an *illness* and regaining a coherent sense of self is a long-standing criticism of the mental health system. These insights inherently refer to underlying assumptions of citizenship. 

### 3.1. Normative Citizenship

There are substantial objections to the idea of individual responsibility “as part of the quest for the model citizen” [45, page 72]. The recovery paradigm can be sharply criticized because of the socially constructed norm of the self-managing, self-sufficient, and independent consumer-citizen who is fully responsible for his/her own choices [24]. A conceptualization of citizenship as normative implies that citizenship is perceived as a status and an achievement [46], mainly based on a norm of active and “good” citizenship that is imposed on individuals and persistently at work in both discourse and practice [23]. In this normative notion of citizenship that promotes “projects of the self” [47], people with mental health problems are expected to become self-sufficient and productive citizens within the scope of self-responsibility, as the responsibility for leading a fulfilling life is individualized [48]. As such, “citizenship becomes conditional on individuals (…) citizens have no rights but responsibilities, and rights shift into social obligations” [23, page 100]. As Rose [49, page 230] observed, “individuals are to become, as it were, entrepreneurs of themselves, shaping their own lives through the choices they make among the forms of life available to them.” The recovery paradigm can be understood against this background, cultivating a project of self-development and self-improvement [47] and enabling societies to make “technologies of opportunity and self-government in the hopes of activating a vital, entrepreneurial and enterprising spirit among (their) subjects” operational [50, page 92]. 

It becomes particularly tricky when this ideology of individual choice and opportunity denies the fact that some citizens have few available choices and resources [46], while at the same time implying that so-called “responsible citizens make reasonable choices and, therefore, ‘bad choices' result from the wilfulness of irresponsible people” [51]. Recovery implies “a danger of running too close to contemporary neoliberal notions of self-help and self-responsibility and glossing over the structural inequalities that hamper personal and social development” [52, page 10]. This logic masks the restricted role of the advanced liberal welfare state [53] in guaranteeing the right to an existence in human dignity, and in pursuing social justice. Although the notion of ideal citizens as choice-making, self-directing, and self-governing subjects in the advanced liberal welfare state is based on individual autonomy and self-responsibility, it lies equally well at the heart of disciplinary control [54, 55]. As Goodley [45, pp. 72-73] argues aptly, a strange paradox emerges for disabled people, including people with mental health problems: “while they are cast as the dependent other, when they do attempt to gain a foothold on the ladder of individualism then they are expected to demonstrate extra-special, hyper-individual forms of being in order to maintain their place (… being) more normal than normal people. (…) And if disabled people fail, then a host of professionals lie in wait to aid and (re)habilitate their journey towards self-containment.” This underlying dynamic refers to the ways in which the provision of care and support is coined by professionals and social services.

### 3.2. Residual Perspective on Care and Support

The recovery paradigm clearly requires a reconceptualization of how social services are (re)organized and delivered [1, 2]. In our view, however, the individual approach to recovery leads easily to residual practices, implying that professionals are expected to empower people with mental health problems in becoming autonomous and self-sufficient citizens, without providing the proper care and support and resources to create fulfilling lives on a structural base. It has been widely observed that minority, marginal, disabled, and chronically ill people might already bear heavy caring responsibilities, but that they also have the fewest social resources and might not be the best risk managers [47]. As citizens, people with mental health problems have the right to be offered care and support, but they do not always fit the support models that make an appeal to the service user's responsibility, “whereby everything would be controlled to the point of self-sustenance, without the need for intervention” [56, page 241]. If the delivery of social services is based on a logic of self-responsibility and self-management of service users with mental health problems, social service professionals might be treading on a tightrope, since they are charged with “motivating and cajoling service users towards projects of autonomy and self-development, while controlling the deviant and destructive aspects of resistance strategies (crime, drugs, benefit fraud, self-harm, mental illness)” [47, page 10]. Social service professionals' preoccupation with empowerment and individual responsibility of service users with mental health problems has been criticized for downplaying and devaluing the provision of care and support [3, 45]. In that light, Rose [53] refers to the privatization of risk, which concerns people who do not, and cannot, live up to the expectations of becoming self-responsible in managing their mental health and other social problems, which creates “a division of the population into those who are capable of managing risk and those whose riskiness requires management under what might be called a tutelary relationship, a division that might be expressed as one between the ‘civilized' and the ‘marginalized'” [57]. As soon as individual citizens cannot prove that they are able to participate in the societal game as self-governing entrepreneurs, they become the objects of intensified surveillance, control, and disciplinary practices [13, 19, 51, 58]. The tendency to transform the responsibility for social risks into a problem of “self-care” inherently contributes to the individualization, decontextualization, and depoliticization of social problems [59].

In that vein, the notion of the ideal citizen marginalizes “competing conceptions of the citizen-subject” [60, page 291] and constructs and transforms some citizens gradually into members of a residual category of nonrecyclable and nondeserving citizens who become waste products in society [61]. Clarke [51, page 453] introduces the conception of the *abandoned citizen*, which unveils “the dynamics of activation, empowerment and responsibilization as rhetorical, masking the real dynamic of abandonment” of residual social practices, in which chiefly an economic rationality is brought to bear on social problems [48]. This residual approach turns social policy into an instrument for rationing services into risk assessment rather than furnishing better care and support, due to scarce resources that are covered under the veil of autonomy, choice, and empowerment [47]. Following this line of thought, the conception of self-managing citizens is a means of reducing costs and pressures on social service systems, as they become “expert patients” and create mutual self-help, take on managing their own lifestyles and well-being, and require less direct attention from residential (and more expensive) services since they learn to embrace the spirit of “do-it-yourself” [51]. The focus lies on the definition of prestructured criteria for access to care and support, and only those “worthy” of care—those who are willing to learn to play the game of self-responsibility—are allowed into the system. Such a vision of humanity threatens to individualize social life, changing individuals rather than society, and fails to support people in their social contexts. From Clarke's [51, page 453] point of view, this version of “responsibility appears as a smokescreen behind which the state is systematically divesting its responsibilities,” including dismantling social services and particularly residential services that are subsidized by the state. Hence, the focus of recovery lies on the characteristics of people with mental health problems, rather than on the policy and organization of the support system [62]. 

## 4. A Social Approach to Recovery

In the extensive body of recovery literature, rather infrequently a social approach to recovery is identified that covers different connotations [2, 8, 10, 63–66]. In embracing the social nature of recovery, of crucial importance is the finding that recovery processes cannot be forced into a cookbook full of recipes for everyone to follow, since recovery often consists of a turbulent process of ups and downs, given the heterogeneous situations of people with mental health problems, implying that “the manifestation and course of their mental illness are unique to them and often non-linear” [11, page 887]. As Ridgway [33, page 339] asserts, “recovery is not linear, the journey is not made up of a specific succession of stages or accomplishments, and it does not follow a straight course. Instead, recovery is an evolving process, one that sometimes spirals back upon itself, and may result in a frustrating return to active disorder.” In that light, Whitwell [63, page 621] refers to *the myth of recovery*, meaning “being restored to your former state (…) as a state of a person, as the end state following a period of illness.” As an exploration of the experiences of people with mental health problems shows that people are conscious of their impaired life position, describing “unemployment, divorce, housing problems, lack of money and social isolation” [63, page 622], a conceptual shift implies moving into a nuanced and *social* understanding of recovery. Also, Tew et al. [10, page 444, our italics] have recently revealed that recovery “emphasises rebuilding a worthwhile life, *irrespective of whether or not one may continue to have particular distress experiences*—and central to this can be reclaiming valued social roles. (…) Recovery may involve a journey both of personal change and of social (re)engagement—which highlights the importance of *creating accepting and enabling social environments within which recovery may be supported*.” Secker et al. [64, page 410, our italics] describe a reconceptualization of recovery that is “viewed as establishing a dynamic and meaningful life *with an impairment* (…), the process of recovery involves the reintroduction of the individual into *a socially accepting and acceptable environment*.” According to Slade [2, page 703], this social approach to recovery can be summarized as “recovery begins when you find someone or something to relate to. The job of the system is to support the relationship (…), maintaining an organizational commitment to recovery, and promoting citizenship among individuals in recovery.” In our view, these insights refer to the necessity to consider notions and interpretations of citizenship in these social practices as relational and inclusive. 

### 4.1. Relational and Inclusive Citizenship

In reality, our societies are often characterized by the dynamics of social exclusion and marginalization [67]. The experience of people with mental health problems of not being recognized as citizens is frequently identified [21, 30–34] and refers to the discrepancy between their formal citizenship (embodied as an entitlement and a status) and their de facto citizenship (constructed through the experience of being a member of a particular community and society in practice) [46]. Lawy and Biesta [68, page 43] refer to a notion of citizenship articulated as relational and inclusive that does not presume that people move through a prespecified trajectory into their citizenship status/achievement as “good” and contributing citizens, yet “it is inclusive rather than exclusive because it assumes that *everyone* in society (…) are citizens who simply move *through* citizenship-as-practice, from the cradle to the grave.” Pols [69] introduced the concept of *relational citizenship*, which differs radically from normative citizenship, as it “develops in the relationship between people, embedded in a set of relational questions, interests and concerns” [23, page 103]. Winance [70] observes that, in practices of citizenship in which normalization processes are challenged from the position of an alignment to work on the norm, the societal norm gets problematized on a collective level. In that vein, inclusive citizenship implies that “the main components of citizenship—membership and belonging, the rights and obligations that flow from that membership, and equality of status—(…) should all apply to all citizens equally” [71, page 4]. In this perspective, citizenship is shaped through relations where norms have to be renegotiated, performed, refreshed, and reestablished in each situation [23]. As such, rights and responsibilities are actualized and constantly renegotiated through (inter)actions in which contradiction and temporary consensus are vital elements [72]. In this frame of reference, the value of care and support depends on the ongoing engagement of professionals in shaping the relationship between the citizen with mental health problems and everyday society as the terrain of interactions with other people, based on an assumption of interdependency and joint responsibility which is redefined in every situation [73]. 

### 4.2. Structural Perspective on Care and Support

According to Beresford and Croft [16], an alliance between service users with mental health problems and professionals is likely to be the most productive way forward for securing the interests of both. Here the question of what care and support mean for people with mental health problems in everyday life plays a pivotal role and requires a continuous dialogue between the client and the professional [23]. Borg and Davidson [73, page 139] stress that supporting people with mental health problems to exercise all of the rights and responsibilities involved in citizenship is the key implication for practice, as “living conditions, income, employment/unemployment, and social interactions outside of treatment settings are central to processes of recovery and cannot be seen as lying outside of the scope of clinical or rehabilitative practice.” In that vein, responsibility might be approached as *the ability to respond* [74], based on the recognition of the fundamental elements of community in which every citizen should have the opportunity to participate: housing, education, income, and work [75].

However, we also want to address implications at the level of social service provision. In a structural perspective on support services, the focus shifts from prestructured criteria of access to the criteria of qualitative social support [76, 77]. These criteria question the ways in which organizations are structured and function in relation to a diversity of clients and problems as well as in relation to those clients and problems that remain off the picture in a residual perspective because they do not manage to become self-sufficient citizens. According to this theoretical frame of reference, five interrelated features need to be constructed as leverages for (more) equality and quality, defined as* availability*,* accessibility*,* affordability*,* usefulness, and comprehensibility *[72].

- Availability refers to the existence of a supply and to the fact that social services can also be called upon for matters that do not necessarily relate directly to the assessed problem.
- Accessibility refers to the (lack of) thresholds when care is needed, for instance an inadequate knowledge of the supply.
- Affordability refers to financial and other costs that the client may encounter, for instance giving up one's privacy or the negative social and psychological consequences of an intervention.
- Usefulness refers to the extent to which the client experiences the care as supportive: is the help attuned to the demand, the skills, and the language of the client?
- Comprehensibility refers to the extent to which clients are aware of the reasons for the intervention and the way in which the problem should be approached.

This implies that the welfare state should develop a differentiated supply of social services that offers all its citizens, in a diversity of situations, the scope to develop their full potential from a structural perspective on care and support [72].

## 5. Conclusion

The concept of recovery can be interpreted against the background of the processes of change in social service systems in many developed countries since the mid-1980s. In this paper, we aimed to explore the pitfalls and the opportunities of the recovery paradigm in relation to these changing service organizations, based on underlying notions of citizenship of people with mental health problems. On the one hand, an individual approach to recovery is identified, undergirded by a neoliberal and normative conception of citizenship, which conceives citizenship as circumscribing the domain of the active entrepreneurial spirit [51]. Those service users with mental health problems who are provided with care and support are committed to act as responsible and reasonably enterprising citizens. In this conception of normative citizenship, these issues are seen as natural, uncontested, and incontestable, and they risk to range people out as nonrecyclable and abandoned citizens [61]. On the other hand, we reclaim a social approach to recovery that implies a conception of relational and inclusive citizenship [22, 23, 70, 71]. This conceptualization of citizenship offers new perspectives for both people with mental health problems and social service professionals, since the debate continues about the actualization of citizenship, about the conditions in which people are expected to lead a dignified life in the community, and about the care and support needed. A high-quality supply of social services that is *made usable *for all its citizens needs to be provided by the welfare state [72].

## References

[1] Slade M (2009). The contribution of mental health services to recovery. *Journal of Mental Health*.

[2] Slade M (2012). Everyday solutions for everyday problems: how mental health systems can support recovery. *Psychiatric Services*.

[3] Beresford P (2010). Re-examining relationships between experience, knowledge, ideas and research: a key role for recipients of state welfare and their movements. *Social Work & Society*.

[4] Beresford P (2010). Public partnerships, governance and user involvement: a service user perspective. *International Journal of Consumer Studies*.

[5] Anthony WA Recovery from mental illness: the guiding vision of the mental health service system in the 1990s. *Psychosocial Rehabilitation Journal*.

[6] Deegan G (2003). Discovering recovery. *Psychiatric Rehabilitation Journal*.

[7] Kristiansen K, Kristiansen K, Traustadóttir R (2004). Madness, badness and sadness: ontology control in ‘mental health land’. *Gender and Disability Research in the Nordic Countries*.

[8] Tew J (2011). *Social Approaches to Mental Distress*.

[9] Le Boutillier C, Leamy M, Bird V, Davidson L, Williams J, Slade M (2011). What does recovery mean in practice? A qualitative analysis of international recovery-oriented practice guidance. *Psychiatric Services*.

[10] Tew J, Ramon S, Slade M, Bird V, Melton J, Le Boutillier C (2012). Social factors and recovery from mental health difficulties: a review of the evidence. *British Journal of Social Work*.

[11] Stanhope V, Solomon P (2008). Getting to the heart of recovery: methods for studying recovery and their implications for evidence-based practice. *British Journal of Social Work*.

[12] Hopton J (2006). The future of critical psychiatry. *Critical Social Policy*.

[13] Davidson G, Campbell J (2007). An examination of the use of coercion by assertive outreach and community mental health teams in Northern Ireland. *British Journal of Social Work*.

[14] Rushton A (1990). Literature review: community-based versus hospital-based care for acutely mentally ill people. *British Journal of Social Work*.

[15] Bartlett P, Wright D (1999). *Outside the Walls of the Asylum: The History of Care in the Community 1750–2000*.

[16] Beresford P, Croft S (2004). Service users and practitioners reunited: the key component for social work reform. *British Journal of Social Work*.

[17] Borg M, Kristiansen K (2004). Recovery-oriented professionals: helping relationships in mental health services. *Journal of Mental Health*.

[18] Postle K, Beresford P (2007). Capacity building and the reconception of political participation: a role for social care workers?. *British Journal of Social Work*.

[19] Wilson G, Daly M (2007). Shaping the future of mental health policy and legislation in Northern Ireland: the impact of service user and professional social work discourses. *British Journal of Social Work*.

[20] Peck E, Gulliver P, Towel D (2002). Information, consultation or control: user involvement in mental health services in England at the turn of the century. *Journal of Mental Health*.

[21] Leamy M, Bird V, Le Boutillier C, Williams J, Slade M (2011). Conceptual framework for personal recovery in mental health: systematic review and narrative synthesis. *The British Journal of Psychiatry*.

[22] Roets G, Kristiansen K, Van Hove G, Vanderplasschen W (2007). Living through exposure to toxic psychiatric orthodoxies: exploring narratives of people with ‘mental health problems’ who are looking for employment on the open labour market. *Disability & Society*.

[23] Roets G, Roose R, Claes L, Vandekinderen C, Van Hove G, Vanderplasschen W (2012). Reinventing the employable citizen: a perspective for social work. *British Journal of Social Work*.

[24] Vandekinderen C, Roets G, Vandenbroeck M, Vanderplasschen W, Van Hove G (2012). One size fits all? The social construction of dis-employ-abled women. *Disability & Society*.

[25] Vandekinderen C, Roets G, Van Hove G Untangling the non-recyclable citizen: a critical reconceptualization of responsibility in recovery.

[26] Chamberlin J (1984). Speaking for ourselves: an overview of the ex-psychiatric inmates' movement. *Psychiatric Rehabilitation Journal*.

[27] Zinman S (1986). Self-help: the wave of the future. *Hospital and Community Psychiatry*.

[28] Lovejoy M (1982). Expectations and the recovery process. *Schizophrenia Bulletin*.

[29] Leete E (1989). How I perceive and manage my illness. *Schizophrenia Bulletin*.

[30] Unzicker R (1989). On my own: a personal journey through madness and re-emergence. *Psychological Rehabilitation Journal*.

[31] Deegan P Recovery and the conspiracy of hope. http://www.patdeegan.com/pat-deegan/lectures/conspiracy-of-hope.

[32] Young SL, Ensing DS (1998). Exploring recovery from the perspective of people with psychiatric disabilities. *Psychiatric Rehabilitation Journal*.

[33] Ridgway P (2001). Restorying psychiatric disability: learning from first person recovery narratives. *Psychiatric Rehabilitation Journal*.

[34] Davidson L (2003). *Living Outside Mental Illness: Qualitative Studies of Recovery in Schizophrenia*.

[35] Ralph RO (2000). Recovery. *Psychiatric Rehabilitation Skills*.

[36] Saleebey D (2009). *The Strengths Perspective in Social Work Practice*.

[37] Borg M, Kristiansen K (2008). Working on the edge: the meaning of work for people recovering from severe mental distress in Norway. *Disability & Society*.

[38] Jacobson N, Greenley D (2001). What is recovery? A conceptual model and explication. *Psychiatric Services*.

[39] Gottstein J (2003). Recovery: responsibilities and roadblocks. *International Center for the Study of Psychiatry and Psychology Newsletter*.

[40] SAMHSA Working Definiton of Recovery: Definition & Components (n.d.). http://www.mhrecovery.com/definition.htm.

[41] Teeple G (1995). *Globalization and the Decline of Social Reform*.

[42] Roberts G, Davenport S, Hollowayand F, Tattan T (2006). *Enabling Recovery: The Principle and Practice of Rehabilitation Psychiatry*.

[43] Chamberlin J (1997). A working definition of empowerment. *Psychiatric Rehabilitation Journal*.

[44] Craig TKJ (2008). Recovery: say what you mean and mean what you say. *Journal of Mental Health*.

[45] Goodley D (2011). *Disability Studies. An Interdisciplinary Introduction*.

[46] Lister R (1997). *Citizenship: Feminist Perspectives*.

[47] Jordan B (2004). Emancipatory Social Work? Opportunity or Oxymoron. *British Journal of Social Work*.

[48] Cruikshank B (1999). *The Will to Empower: Democratic Citizens and Other Subjects*.

[49] Rose N (1989). *Governing the Soul: the Shaping of the Private Self*.

[50] Binkley S (2011). Psychological life as enterprise: social practice and the government of neo-liberal interiority. *History of the Human Sciences*.

[51] Clarke J (2005). New Labour’s citizens: activated, empowered, responsibilized, abandoned?. *Critical Social Policy*.

[52] Gray M (2011). Back to basics: a critique of the strengths perspective in social work. *Families in Society*.

[53] Rose N (1993). Government, authority and expertise in advanced liberalism. *Economy and Society*.

[54] Miller P, Rose N (2004). *Governing the Present: Administering Economic, Social and Personal Life*.

[55] McNay L (2009). Self as enterprise: dilemmas of control and resistance in foucault’s the birth of Biopolitics. *Theory, Culture & Society*.

[56] Foucault M, Rabinow P (1984). Space, knowledge and power. *The Foucault Reader*.

[57] Dean M (1995). Governing the unemployed self in an active society. *Economy & Society*.

[58] Jordan B, Jordan C (2000). *Social Work and the Third Way: Tough Love as Social Policy*.

[59] Lemke T (2001). ’The birth of bio-politics’: Michel Foucault’s lecture at the Collège de France on neo-liberal governmentality. *Economy and Society*.

[60] Foucault M (2008). *The Birth of Biopolitics: Lectures at the Collège de France 1978-1979*.

[61] Ledoux M (2004). *What Are We Doing? Institutional Psychotherapy in Resistance and Dialogue with Quality Psychiatry*.

[62] Roose R (2006). *Child Welfare and Protection as Educator*.

[63] Whitwell D (1999). The myth of recovery from mental illness. *Psychiatric Bulletin*.

[64] Secker J, Membrey H, Grove B, Seebohm P (2002). Recovering from illness or recovering your life? Implications of clinical versus social models of recovery from mental health problems for employment support services. *Disability & Society*.

[65] Mezzina R, Davidson L, Borg M, Marin I, Topor A, Sells D (2006). The social nature of recovery: discussion and implications for practice. *American Journal of Psychiatric Rehabilitation*.

[66] Slade M, Williams J, Bird V, Leamy M, Le Boutillier C (2012). Recovery grows up. *Journal of Mental Health*.

[67] Kabeer N (2005). *Inclusive Citizenship: Meanings and Expressions*.

[68] Lawy R, Biesta G (2006). Citizenship-as-practice: the educational implication of an inclusive and relation understanding of citizenship. *British Journal of Educational Studies*.

[69] Pols J (2006). Washing the citizen: washing, cleanliness and citizenship in mental health care. *Culture, Medicine and Psychiatry*.

[70] Winance M (2007). Being normally different? Changes to normalization processes: from alignment to work on the norm. *Disability & Society*.

[71] Lister R (2008). Inclusive citizenship, gender and poverty: some implications for education for citizenship. *Citizenship Teaching and Learning*.

[72] Roose R, De Bie M (2003). From participative research to participative practice—a study in youth care. *Journal of Community and Applied Social Psychology*.

[73] Borg M, Davidson L (2008). The nature of recovery as lived in everyday experience. *Journal of Mental Health*.

[74] Newbury A (2008). Youth crime: whose responsibility?. *Journal of Law and Society*.

[75] Teghtsoonian K (2009). Depression and mental health in neoliberal times: a critical analysis of policy and discourse. *Social Science and Medicine*.

[76] Hubeau B, Parmentier S (1991). Preadvies rechtshulp. *Aanbevelingen voor het armoedebestrijdingsbeleid, 1990-1991: derde verslag van de Interdepartementale Commissie voor de Armoedebestrijding*.

[77] Hubeau B, Parmentier S (2008). Sociale rechtshulp: algemene ontwikkeling. *Welzijnsgids*.

